# Reconstruction of stented coronary arteries from optical coherence tomography images: Feasibility, validation, and repeatability of a segmentation method

**DOI:** 10.1371/journal.pone.0177495

**Published:** 2017-06-02

**Authors:** Claudio Chiastra, Eros Montin, Marco Bologna, Susanna Migliori, Cristina Aurigemma, Francesco Burzotta, Simona Celi, Gabriele Dubini, Francesco Migliavacca, Luca Mainardi

**Affiliations:** 1 Laboratory of Biological Structure Mechanics (LaBS), Department of Chemistry, Materials and Chemical Engineering “Giulio Natta”, Politecnico di Milano, Milan, Italy; 2 Department of Electronics, Information and Bioengineering, Politecnico di Milano, Milan, Italy; 3 Institute of Cardiology, Catholic University of the Sacred Heart, Rome, Italy; 4 Fondazione Toscana G. Monasterio, Ospedale Del Cuore, Massa, Italy; Technion Israel Institute of Technology, ISRAEL

## Abstract

Optical coherence tomography (OCT) is an established catheter-based imaging modality for the assessment of coronary artery disease and the guidance of stent placement during percutaneous coronary intervention. Manual analysis of large OCT datasets for vessel contours or stent struts detection is time-consuming and unsuitable for real-time applications. In this study, a fully automatic method was developed for detection of both vessel contours and stent struts. The method was applied to *in vitro* OCT scans of eight stented silicone bifurcation phantoms for validation purposes. The proposed algorithm comprised four main steps, namely pre-processing, lumen border detection, stent strut detection, and three-dimensional point cloud creation. The algorithm was validated against manual segmentation performed by two independent image readers. Linear regression showed good agreement between automatic and manual segmentations in terms of lumen area (r>0.99). No statistically significant differences in the number of detected struts were found between the segmentations. Mean values of similarity indexes were >95% and >85% for the lumen and stent detection, respectively. Stent point clouds of two selected cases, obtained after OCT image processing, were compared to the centerline points of the corresponding stent reconstructions from micro computed tomography, used as ground-truth. Quantitative comparison between the corresponding stent points resulted in median values of ~150 μm and ~40 μm for the total and radial distances of both cases, respectively. The repeatability of the detection method was investigated by calculating the lumen volume and the mean number of detected struts per frame for seven repeated OCT scans of one selected case. Results showed low deviation of values from the median for both analyzed quantities. In conclusion, this study presents a robust automatic method for detection of lumen contours and stent struts from OCT as supported by focused validation against both manual segmentation and micro computed tomography and by good repeatability.

## 1. Introduction

Intravascular optical coherence tomography (OCT) is an established catheter-based imaging modality for the assessment of coronary artery disease and the guidance of percutaneous coronary interventions [1–3]. This imaging technique uses a near-infrared laser source (~1310 nm) to target the vessel wall and produces cross-sectional vessel images by elaborating the intensity of the interferometric signal that is generated by the light reflected from the sample [4].

Compared to other imaging techniques for coronary arteries, such as angiography, computed tomography, magnetic resonance imaging, and intravascular ultrasound, OCT is characterized by higher resolution and the possibility to detect both the vessel lumen and the implanted stents [3]. In particular, current OCT systems provide an axial and lateral resolution of 12–15 μm and 20–40 μm, respectively, and a penetration depth in the arterial wall of 1–2.5 mm [5]. These features make OCT suitable for the assessment of atherosclerotic lesions by quantifying the extension of lumen narrowing and by allowing the visualization of atherosclerotic plaques, in terms of composition and shape [6]. For instance, lipid, fibrotic, or calcified plaques, as well as thrombi, can be identified with this imaging modality [6]. Furthermore, OCT characteristics ensure the guidance of percutaneous coronary intervention, providing information on vessel size and stent strut apposition [3]. OCT can be also used to identify the signs of vessel trauma, such as dissections or tissue prolapse, immediately after stent implantation [6]. Additionally, OCT allows the evaluation of tissue coverage in follow-up analyses [7].

Automated methods for the analysis of OCT images are currently available for research purposes, even though a standardization for their validation and application is absent [3]. Over the last years, several methods have been proposed to analyze OCT datasets in order to automatically detect the lumen contours [8–22] and stent struts [9,10,16–18,20,22–27]. In general, those automatic algorithms showed good results with respect to manual analysis. However, algorithms for the identification of both lumen contours and stent struts, able to take into account the issues related to the presence of bifurcation branches, were reported only in few studies [10,16]. Moreover, validation of these algorithms was performed only against manual segmentation and their repeatability was not evaluated.

In this work, a fully automatic detection and three-dimensional (3D) visualization method of both lumen contours and stent struts from OCT imaging of coronary bifurcations is presented. This method was applied to *in vitro* OCT datasets that were acquired in stented silicone bifurcation phantoms. The proposed methodology was validated against both manual segmentation and micro computed tomography (micro-CT) 3D reconstructions. Furthermore, the repeatability of the detection algorithm was assessed by evaluating results obtained with the proposed procedure for repeated OCT scans of the same case. Finally, the method was applied to *in vivo* OCT datasets to demonstrate its applicability to patient-specific cases.

Applications of the proposed method include the quantification of lesion severity and stent strut malapposition, and the creation of 3D models of stented vessels to perform computational fluid dynamics simulations for the analysis of the local hemodynamics in the microenvironment of the stent struts.

## 2. Material and methods

### 2.1 Coronary bifurcation phantoms and stents

**Fig 1:**
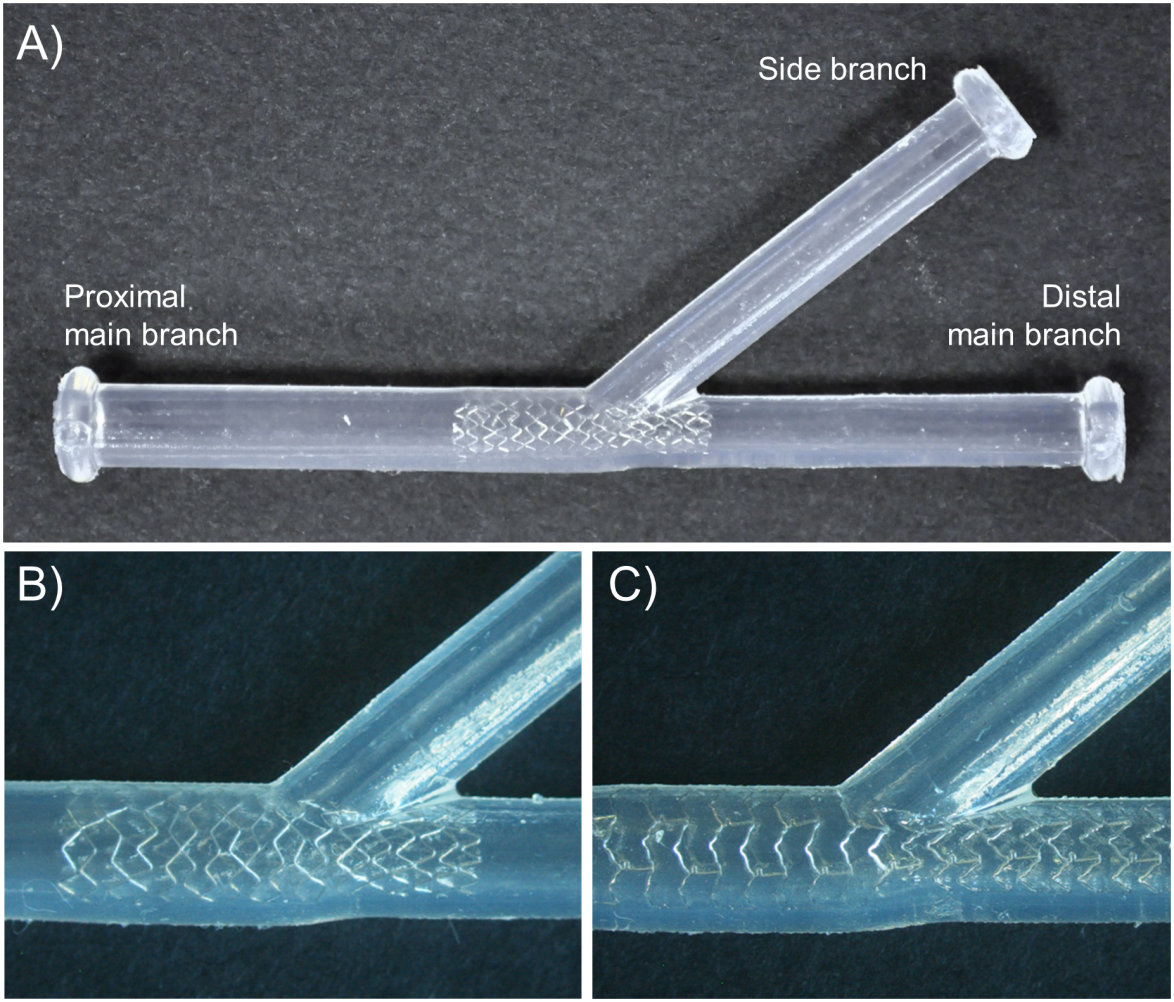
Examples of stented coronary bifurcation phantoms: A) Bifurcation phantom with a bifurcation angle of 40° and a Resolute Integrity stent (Case 1). B) Detail of the Resolute Integrity stent implanted in a 40° bifurcation phantom (Case 1). C) Detail of the Xience Prime stent implanted in a 40° bifurcation phantom (Case 2).

Eight coronary bifurcation phantoms were fabricated through a casting process using polydimethylsiloxane (PDMS—Sylgard 184) (Fig 1A). The phantoms were planar with straight branches, constant thickness and lumen diameters. The bifurcation angle and diameters of each phantom (Table 1) were in the physiological range for coronary bifurcations [28]. Additionally, the diameters obeyed Finet’s law [29].

**Table 1:** Main characteristics of the coronary bifurcation phantoms, implanted stent types, and stenting procedures followed by interventional cardiologists for stent implantation.

ID	Diameter [mm]			Bifurcation angle [°]	Mixture ratio (base:cross-linker)	Stent type	Stenting technique
	PMB	DMB	SB				
1	3.8	2.9	2.75	40	5:1	Resolute Integrity 3x18 mm	PSB
2	3.8	2.9	2.75	40	5:1	Xience Prime 3x28 mm	PSB
3	3.5	2.76	2.4	45	5:1	Resolute Integrity 3x26 mm	PSB
4	3.5	2.76	2.4	45	5:1	Resolute Integrity 3x26 mm	PSB + KBI
5	3.5	2.76	2.4	45	15:1	Resolute Integrity 3x26 mm	PSB
6	3.5	2.76	2.4	45	15:1	Resolute Integrity 3x26 mm	PSB+ KBI
7	3.5	2.76	2.4	45	25:1	Resolute Integrity 3x26 mm	PSB
8	3.5	2.76	2.4	45	25:1	Resolute Integrity 3x26 mm	PSB+ KBI

ID—bifurcation identifier; PMB—proximal main branch; DMB—distal main branch; SB—side branch; PMB—provisional side branch stenting technique; KBI—kissing balloon inflation

Commercially available coronary stents were implanted in the phantoms by an interventional cardiologist, following common interventional procedures. In particular, seven zotarolimus eluting stents (ZES, Resolute Integrity Medtronic, Minneapolis, MN, USA) (Fig 1B) and one everolimus eluting stent (EES, Xience Prime, Abbott Vascular, Santa Clara, CA, USA) (Fig 1C) were deployed. The provisional side branch stenting approach is the most commonly used procedure to treat bifurcation coronary lesions [30]. According to this technique, a stent is implanted in the main branch and an optional treatment of the side branch is considered in case of sub-optimal side branch result after main branch stenting. In our cases, an “optimized” provisional stent technique was performed [31]. Briefly, a stent was implanted in the main branch and then post-dilatation was performed with a short balloon at the stent proximal segment (up to the carina level) (i.e. proximal optimization technique, POT) to avoid proximal malapposition while limiting carina shift and distal edge dissections. In three cases, kissing balloon inflation (KBI) (i.e. simultaneous dilation of two balloons, sizes 1:1 according to side branch and distal main branch diameters) was also carried out after main branch stenting and POT to improve side branch access and prevent stent distortion within the main branch [30]. A final re-POT was performed to reduce the elliptical deformation of the stent in the proximal main branch after KBI. The stent type and size, and the stenting procedure used for each bifurcation phantom are reported in Table 1.

### 2.2 Image data collection

OCT images of the main branch of the bifurcation phantoms were acquired in DICOM format using the commercially available Fourier-Domain OCT system C7-XR (St. Jude Medical, St. Paul, MN, USA) with a C7 Dragonfly catheter (St. Jude Medical). For each bifurcation phantom one OCT pullback procedure was performed, except for case 3, for which the pullback was repeated seven times to verify the repeatability of the procedure. The bifurcation phantoms were immersed into water at room temperature to enhance image quality. The automated pullback speed was 18 mm/s with a data frame rate of 180 frames per second and a pullback length of 54 mm. A set of 540 cross-sectional images was acquired during each OCT scan with axial and lateral resolutions of 12–18 μm and 20–90 μm, respectively, and a distance between frames of 100 μm.

Micro-CT of two bifurcation phantoms with different implanted stents (i.e. Case 1, with Resolute Integrity stent, and Case 2, with Xience Prime stent) was also performed to validate the stent detection algorithm from OCT images. The Xalt micro-CT scanner, equipped with a W-anode microfocus X-ray source with accelerating potential in the range of 20–50 kV and with a 10×5 cm^2^ flat-panel CMOS detector with Gadox scintillator, was used [32]. An isotropic voxel of 18 μm was obtained. The micro-CT slices were processed in Mimics (Materialise, Leuven, Belgium), which allowed discretization of the stent and computation of its centerline.

### 2.3 OCT image processing

The collected OCT data were analyzed off-line using an automatic image processing procedure implemented in Matlab (Mathworks, Natick, MA, USA). This procedure comprises four steps: pre-processing, lumen edge detection, stent-strut detection, and 3D point cloud creation.

#### 2.3.1 Pre-processing

**Fig 2:**
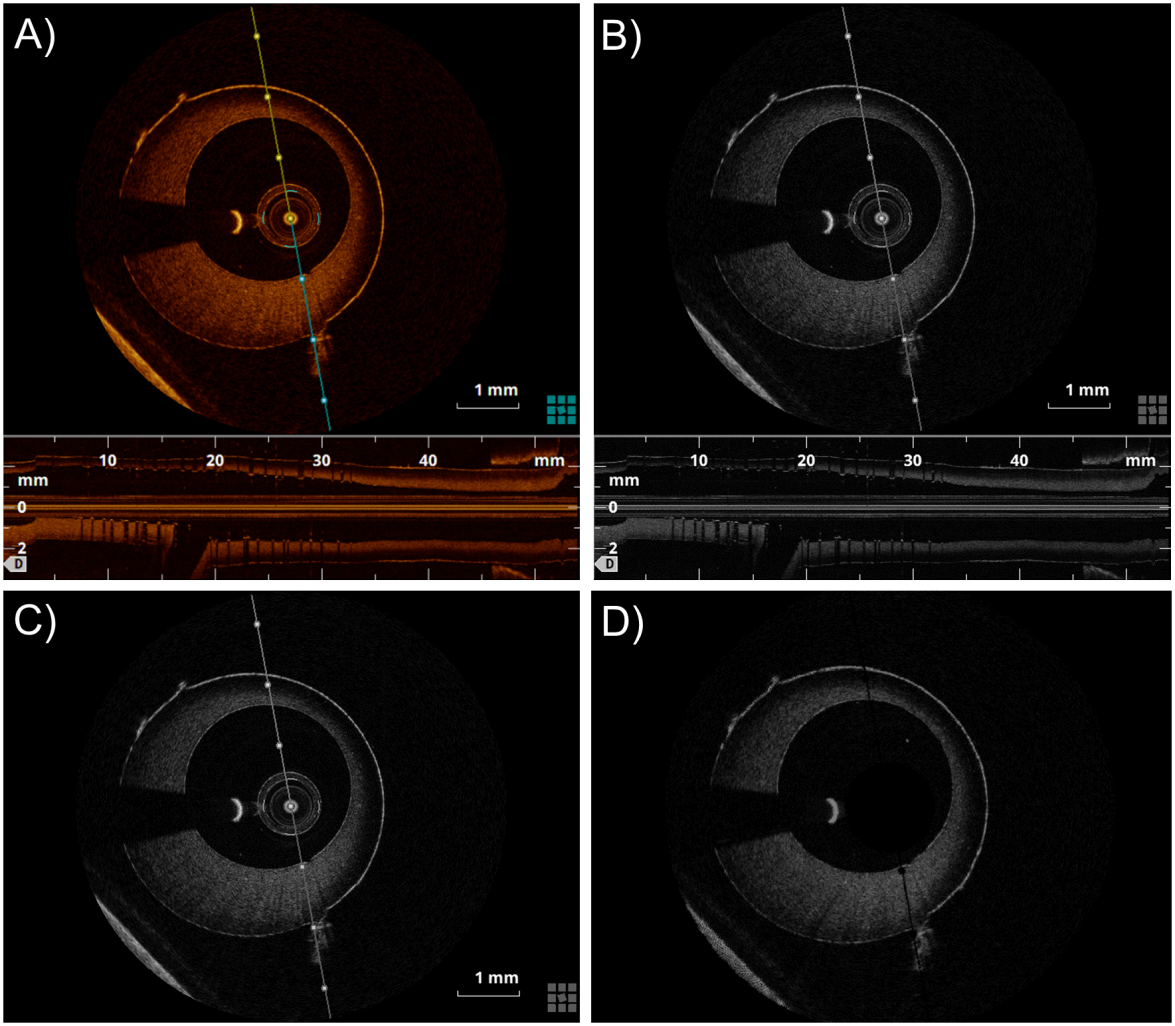
Pre-processing steps: A) Original RGB OCT image. B) Greyscale image. C) Image after crop of the lower part, which represents the longitudinal view of the vessel phantom. D) Image without visualization tools and catheter.

**Fig 3:**
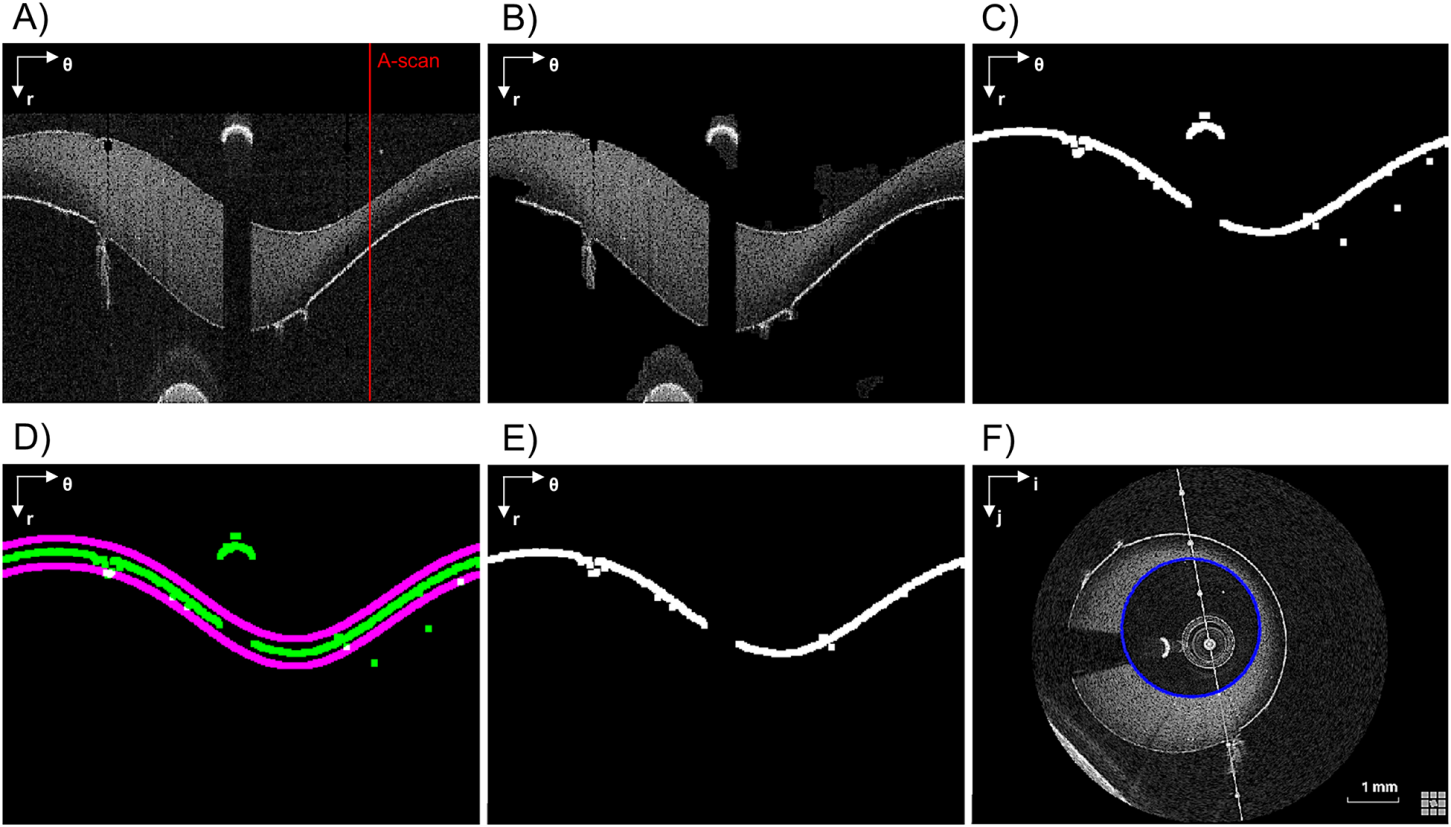
Lumen contour detection steps. A) Pre-processed image (in polar coordinates). The red line highlights an example of A-scan. B) Image without background noise. C) Raw lumen contour detection. D) Detected lumen contour (green) and validity region of the segmentation (purple). E) Lumen contour without misdetections. F) Lumen contour (blue) detected after gaps closing, smoothing, and conversion back to Cartesian coordinates. The polar coordinate system (r; θ) or the Cartesian coordinate system (i; j) is indicated on the top left of each image.

The pre-processing step is necessary to prepare the OCT images (Fig 2A) for segmentation. The algorithm elaborates each frame in sequence, thus the following procedure is repeated for all frames. First, the OCT frame is converted from RGB to greyscale (Fig 2B). Second, the lower part of the frame, which represents the longitudinal view of the vessel phantom, is cropped. Third, elements that are related to the OCT visualization tools (i.e. line representing the section plane of the longitudinal section, scale, and other information related to the image) are removed by observing that their position is the same across all frames (Fig 3C). Therefore, the intensity of their pixels are set to background value. Lastly, an average is computed across frames to remove the cross-section of the OCT catheter. Since the catheter cross-section is always in the same position across frames, it is not affected by averaging while other structures such as the vessel border are reduced as they may change position frame by frame. Thus, the catheter results in the highest pixel value after the average and can be removed by thresholding procedure. Fig 2D shows the final result of the pre-processing step on a single OCT frame.

#### 2.3.2 Lumen border detection

To identify the lumen border, image binarization is first performed to highlight the higher intensity regions. Then salt-and-pepper noise is reduced by applying a morphological opening (disk of radius 3 pixels as structuring element) followed by an area thresholding. The image is then converted to polar coordinates (r; θ) and the lumen border is identified by means of a Sobel edge detection filter. Due to image noise this border might not be unique in each A-scan (i.e. one-dimensional depth profile, Fig 3A), as at this level we consider as border the first non-zero intensity value of the column.

For each consecutive frame, the border identification in the n-th frame is obtained by considering a validity region built around the lumen border of the previous (n-1)-th slice. This region is centered on the previous lumen border and is 10 pixels wide (purple lines in Fig 3D). All points outside these regions are considered as artifact and are removed. The width of the validity region was empirically chosen to preserve the lumen border while removing the artifacts. It is worth noticing that this last step is not performed on the first slice whose lumen border comes out from the Sobel filter. Finally, in order to fill the gap obtained after the false positive correction (Fig 3E), the border points are smoothed with a moving average filter and linearly interpolated in polar coordinates (Fig 3F).

Usually, the linear interpolation of these gaps and the following smoothing are enough to get an acceptable shape of the lumen border. However, when these errors are widely spread along the θ-coordinate, the error removal algorithm leaves large gaps in the lumen contour, and the linear interpolation fails to fill such large gaps. Therefore, if the gap width is greater than a threshold (i.e. 50° along θ), the gaps are filled with the points of the lumen border identified in the previous slice; then, a smoothing filter with a span of 10% is applied to remove any possible discontinuity. It is worth noting that the presence of errors in a wide range of θ is characteristic of the presence of a bifurcation and this feature can be used to automatically detect the presence of a bifurcation.

#### 2.3.3 Stent struts detection

**Fig 4:**
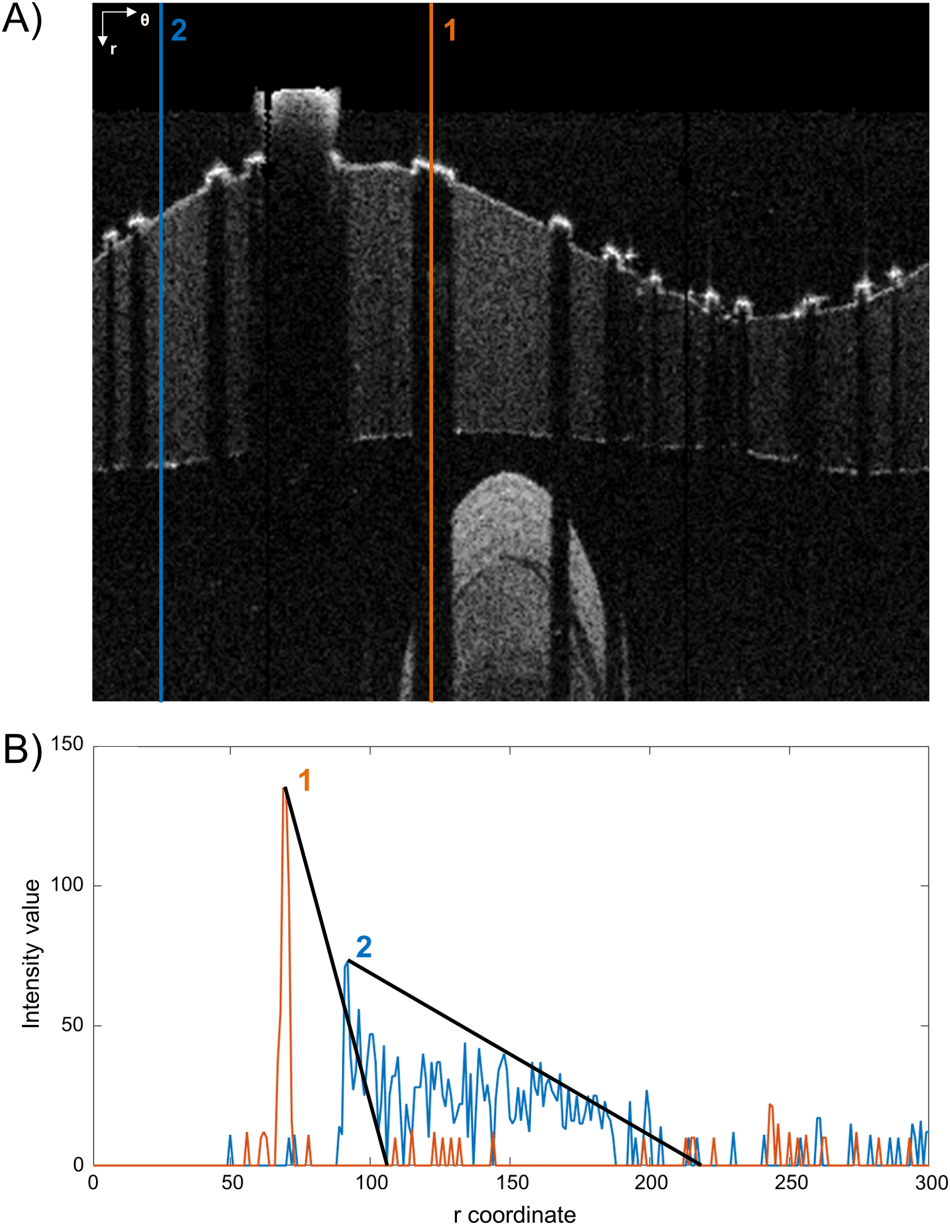
Example of stent strut detection. A) Two A-scans are analyzed. The first one passes through a stent strut while the second one passes only through the vessel wall. The polar coordinate system (r; θ) is indicated on the top left. B) Corresponding intensity profiles of A-scans 1 and 2. The strut is detected because of the higher slope of the intensity profile of its A-scan.

A strut appears in OCT images as a high reflecting zone (high intensity region) accompanied by a trail shadow (low intensity column in polar coordinates) [33]. The detection algorithm searches these features as proposed by Wang and colleagues [24]. In particular, for each A-scan the slope of the line connecting a high intensity peak (i.e. above the 90^th^ percentile of the intensity histogram of the frame) and the following 30^th^ pixel with low intensity (i.e. below the median of the intensity histogram of the frame) is computed. If the line slope is lower than a threshold (-1.5 intensity/pixel), then the peak is considered part of a strut. In case the 30^th^ low intensity pixel is not present because the strut is close to the border of the image domain, a zero padding is performed at the lowest part of the image. Fig 4 shows an example of an A-scan profile with (red line) and without (blue line) strut: the slope of the red line (strut) is higher than that of the blue one (no strut).

The amount of light reflected back from the OCT system is function of a physical parameter called attenuation coefficient, which varies within the sample as a function of the distance from the catheter [34]. Theoretically, areas far from catheter should have lower intensities, but the presence of the outer wall of the vessel and noise might induce false positive detections and worsen the accuracy of Wang’s method. Hence, the A-scan intensity profile was multiplied with a triangular-shaped window which has the maximum around the expected strut position and decreases when the distance from the maximum increases. This penalizes detection of structures far from the lumen border.

**Fig 5:**
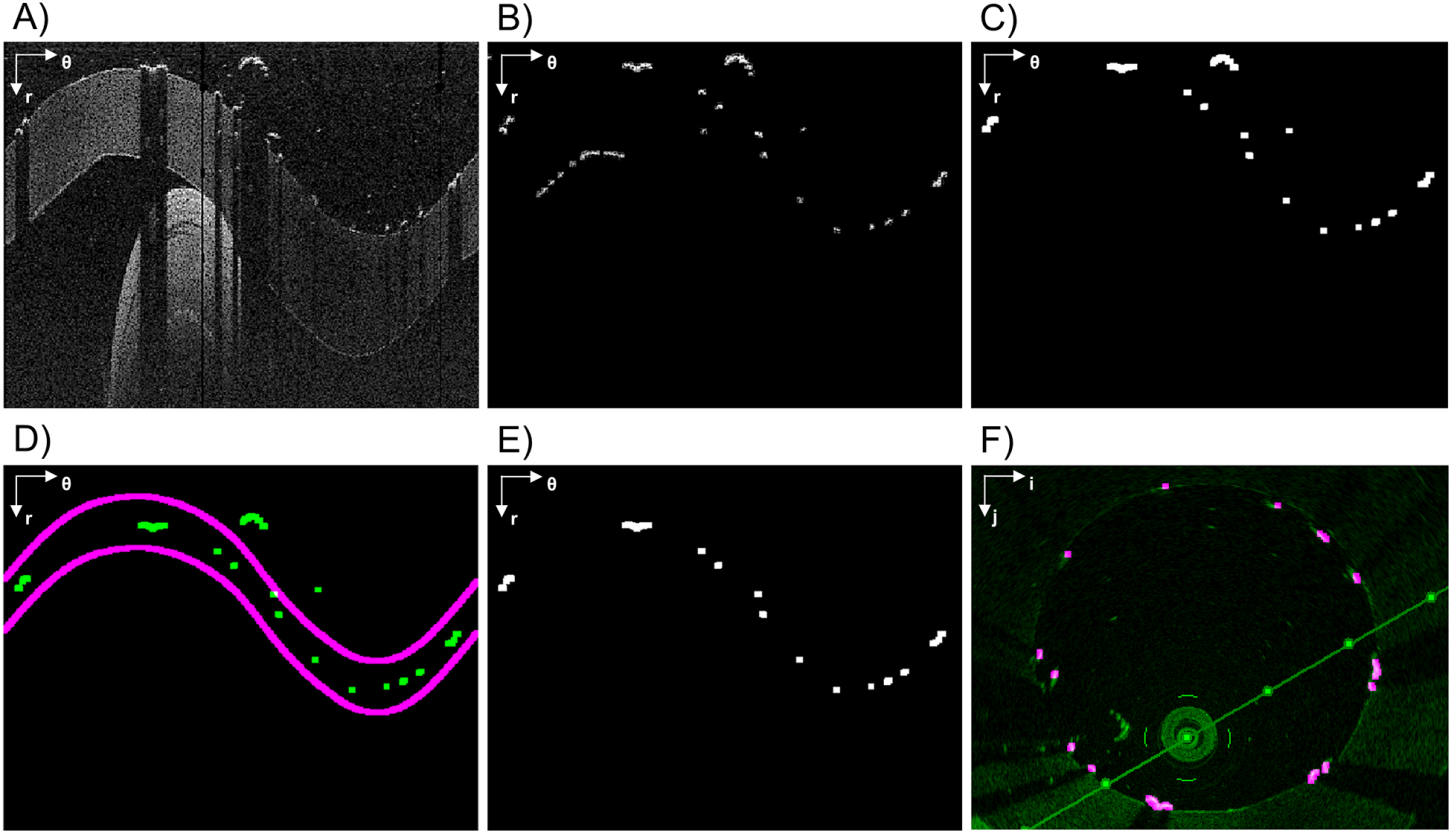
Stent struts detection algorithm steps. A) Pre-processed image (in polar coordinates). B) Rough detection. C) Result of the application of the triangular shaped window followed by an intensity thresholding. D) Detected struts (green) and the validity region of the segmentation (purple). E) Image without errors. F) Detected struts (purple) overlapped to the original image (green) in Cartesian coordinates. The polar coordinate system (r; θ) or the Cartesian coordinate system (i; j) is indicated on the top left of each image.

To avoid misdetection in the current frame, positions of struts in the previous and next five slices are used to define a region of confidence in which the struts are expected to fall. If a strut not falling in that region is identified, it will be removed because considered as an artifact (as for the case of reflection of infrared light on the guide wire or saturation artifacts [35]). An example of the stent strut detection steps is shown in Fig 5. For each identified (and confirmed) strut, the center of mass is finally computed.

#### 2.3.4 Point cloud creation

**Fig 6:**
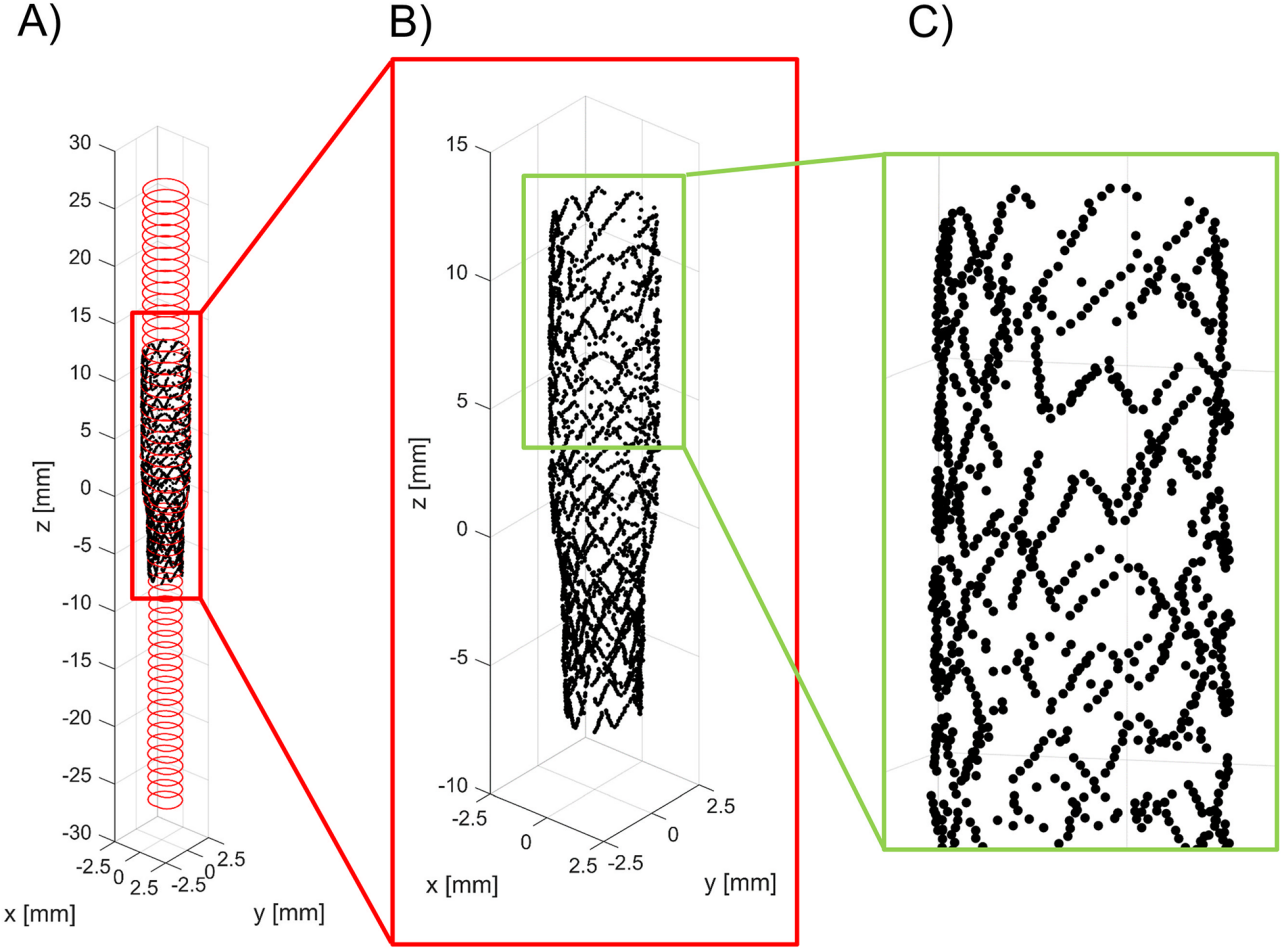
A) Three-dimensional point cloud of the main branch of a bifurcation phantom with an implanted Resolute Integrity stent (case 1) obtained with the lumen border and stent struts detection algorithms. B, C) Details of the stent point cloud.

The detected lumen borders and stent strut centers of mass of each OCT frame are placed orthogonal to the main vessel centerline of the bifurcation phantoms by using the centroid of each lumen border. The vessel centerline is assumed as perfectly straight because the main vessel was fabricated as a straight tube. The inter-frame distance is used to stack the OCT frames. Subsequently, lumen borders and stent struts are represented in 3D, generating a point cloud view (Fig 6).

### 2.4 Validation of the segmentation algorithm

#### 2.4.1 Lumen detection

Results of the lumen detection algorithm were compared with a manual segmentation performed with the open-source software MRIcro (University of South Carolina, Columbia, SC, USA) by two independent expert image readers (R1 and R2) on a randomly selected subset of 160 images (20 images per bifurcation case, with the condition that adjacent frames were excluded).

The lumen area was calculated for each segmented image. A Kruskal-Wallis test was performed to verify the absence of significant differences between the lumen area of each image obtained with the automatic and the two manual segmentations. A value p>0.05 indicated that significant differences between lumen areas were not present. Additionally, linear regression and Bland-Altman analysis [36] were used to assess the agreement between area values obtained with the automatic and the two manual segmentation procedures and to evaluate the inter-observer variability of the manual detections.

In general, the lumen area is insufficient to evaluate the quality of the lumen detection because same area values that were obtained with automatic and manual method might correspond to different lumen border shapes. Thus, for each selected image, pixels defined as lumen border by the two methods were superimposed and used to compute the number of true positives (TP), false positives (FP), false negatives (FN), and true negatives (TN) using as reference the manual segmentation. The following similarity indexes were calculated:

$$Sensitivity=\;\frac{TP}{TP+FN}\;\cdot \;100$$ (1)

$$Specificity=\;\frac{TN}{FP+TN}\;\cdot \;100$$ (2)

$$Jaccard\;index=\;\frac{TP}{TP+FP+FN}\;\cdot \;100$$ (3)

$$Dice\;index=\;\frac{2\;\cdot \;TP}{2\;\cdot \;TP+FP+FN}\;\cdot \;100$$ (4)

Finally, the distribution of the distance between the lumen contours obtained with the automatic and manual segmentations was determined by calculating the absolute value of the distance between each corresponding lumen border point, in polar coordinates (i.e. points belonging to the same A-scan).

All statistical analyses were performed in Matlab.

#### 2.4.2 Stent strut detection

**Automatic versus manual segmentation**

The stent struts detection algorithm was validated by comparing results obtained with a manual detection of struts performed by two trained cardiologists using MRIcro on the same subset of images described in the previous section.

The number of struts detected per frame by the automatic and manual methods was calculated. A Kruskal-Wallis test was conducted to verify the absence of significant differences between the number of identified struts by the two methods. A value p>0.05 indicated that significant differences were not present.

The corresponding OCT frames, which were segmented with both methods, were superimposed and the number of struts detected by both methods (TP), the struts detected only by the automatic method (FP), and the struts not identified by the automatic method (FN) were calculated by considering as reference the manual segmentation. The sensitivity, Jaccard index, and Dice index were computed using the formula (1), (3), and (4), respectively. The specificity was calculated as:

$$Specificity=\;\left(1-\frac{FP}{n_{strut}}\right)\;\cdot \;100$$ (5)

where *n*_*strut*_ is the number of struts detected with the manual segmentation.

The total and radial distances between the centroid of each automatically segmented strut and the nearest manually identified strut was evaluated in order to quantify the differences between struts that were detected with both automatic and manual methods.

Finally, the length of appositions (LOA), defined as the radial distance between a strut and the lumen border [17], was determined in both automatically and manually segmented OCT frames. The agreement between the measurements was assessed through Bland-Altman analysis [36].

All statistical analyses were performed in Matlab.

**Comparison with micro**-**CT**

The stent point clouds of cases 1 and 2, which were obtained by applying the stent struts detection algorithm, were compared in 3D with the centerline points of the same stent reconstructed with micro-CT. Each point cloud was registered to the corresponding centerline points obtained from micro-CT.

The registration was performed in Matlab using the iterative closest point (ICP) algorithm [37], a well-known algorithm for rigid (rotation and translation) registration of 3D point sets, which iteratively minimizes the sum of the squared distances between adjacent points. The micro-CT point cloud was referred as the fixed one while the OCT point cloud as the moving one. The registration was initialized by aligning the OCT point cloud barycenter on that of the micro-CT, which had been chosen as the center of rotation for the rotation part of the transformation model. The ICP optimization was repeated by varying the initialization angle on the z-axis (pullback axis) from 0 to 360 degrees to obtain the optimal transformation which minimizes the squared distances between adjacent points. Finally, the total and radial distances between corresponding points of the OCT and micro-CT stent point clouds were calculated.

### 2.5 Repeatability of the OCT pullback

To test the repeatability of the lumen border detection algorithm, the lumen volume of each case was calculated as the sum of the lumen area per frame multiplied by the distance between the slices. The extremes of the stent were used as landmarks to establish the same region of interest between acquisitions. Regarding the strut detection algorithm, the mean of the number of detected struts per frame was computed for each case.

### 2.6 Applicability to patient-specific cases

Four *in vivo* OCT pullbacks of stented coronary artery segments were retrospectively selected to demonstrate the applicability of the developed algorithms to patient-specific cases. The patients were treated at the Institute of Cardiology, Catholic University of the Sacred Heart (Rome, Italy) with the Resolute Integrity (n = 2) or the Xience Prime (n = 2) stents. OCT images were collected using the same OCT system and acquisition settings as done for the *in vitro* scans. The algorithms of lumen border and stent struts detection were applied using the same parameters as defined for the *in vitro* cases. The analyses were approved by the Ethics Committee of the Catholic University of the Sacred Heart and conformed to the Declaration of Helsinki on human research. All patients gave informed consent.

## 3. Results

### 3.1 Lumen detection

Table 2 reports the 25^th^, 50^th^, and 75^th^ percentiles of the distributions of lumen areas of the OCT images analyzed with the automatic segmentation method and by the two manual readers. No significant differences were found between the lumen areas calculated with the different methods (chi-square = 0.11, p = 0.95).

**Table 2:** Percentiles of the distributions of lumen areas and distance between the lumen contours obtained with the automatic and manual segmentation methods.

	25^th^ percentile	50^th^ percentile	75^th^ percentile
**Lumen area [mm**^**2**^**]**			
** Auto**	5.836	8.608	9.078
** R1**	5.895	8.542	9.032
** R2**	5.875	8.546	9.074
**Lumen contours distance [μm]**			
** Auto vs. R1**	0.0	13.0	26.0
** Auto vs. R2**	13.0	13.0	39.0
** R1 vs. R2**	0.0	13.0	26.0

Auto—automatic detection algorithm; R1 –image reader 1; R2 –image reader 2

**Fig 7:**
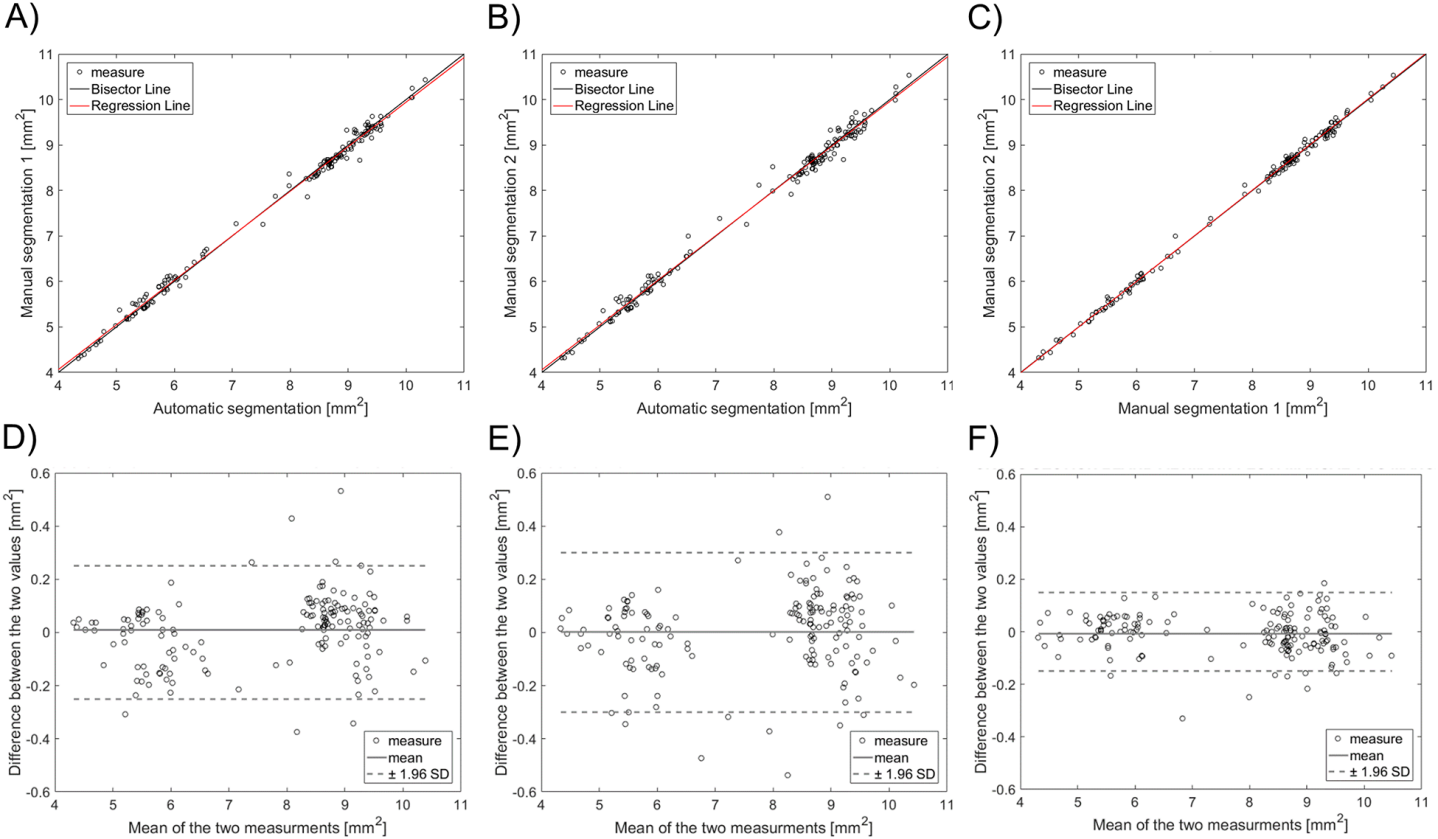
Top—Linear regression plots of the lumen area of 160 randomly selected OCT images: A) automatic segmentation against manual segmentation by image reader 1 (R1); B) automatic segmentation against manual segmentation by image reader 2 (R2); C) manual segmentation by R1 against that by R2. Bottom—Bland-Altman plots of the lumen area: D) automatic segmentation against R1; E) automatic segmentation against R2; F) manual segmentation by R1 against R2.

Linear regression showed a good agreement between automatic and manual segmentations for the assessment of the lumen area (Fig 7A and 7B), resulting in a correlation coefficient of 0.997 (p<0.005) and 0.996 (p<0.005) for readers R1 and R2, respectively. Inter-observer variability of the manual detections (Fig 7C) had a high correlation coefficient (r = 0.999, p<0.005). The Bland-Altman diagrams of the lumen area differences are shown in Fig 7D–7F. The 95% confidence range in the lumen area percentage differences was -3.46% and 3.52%, -4.17% and 4.08%, and -2.11% and 1.96% for the automatic algorithm versus R1, the automatic algorithm versus R2, and R1 versus R2, respectively. The lumen area differences were between -0.25 mm^2^ and 0.27 mm^2^, -0.28 mm^2^ and 0.32 mm^2^, and -0.17 mm^2^ and 0.15 mm^2^ for the automatic algorithm versus R1, the automatic algorithm versus R2, and R1 versus R2, respectively.

The similarity indexes, which are related to the superimposition of the lumen pixels detected with both automatic and manual segmentation methods, are reported in Table 3.

**Table 3:** Similarity indexes of lumen and stent strut detection algorithms.

	Sensitivity	Specificity	Jaccard index	Dice index
**Lumen area**				
**Auto vs. R1**	98.69 ± 1.07%	99.83 ± 0.15%	97.38 ± 1.11%	98.67 ± 0.58%
**Auto vs. R2**	98.25 ± 1.30%	99.76 ± 0.16%	96.59 ± 1.19%	98.26 ± 0.62%
**R1 vs. R2**	98.76 ± 0.64%	99.86 ± 0.01%	97.62 ± 0.56%	98.80 ± 0.29%
**Stent struts**				
**Auto vs. R1**	90.87 ± 9.44%	94.75 ± 7.60%	86.66 ± 10.08%	92.53 ± 5.97%
**Auto vs. R2**	91.27 ± 9.34%	94.69 ± 7.54%	87.00 ± 10.05%	92.73 ± 6.00%
**R1 vs. R2**	98.68 ± 3.73%	98.49 ± 4.54%	97.38 ± 5.28%	98.6 ± 2.93%

Auto—automatic detection algorithm; R1 –image reader 1; R2 –image reader 2

**Fig 8:**
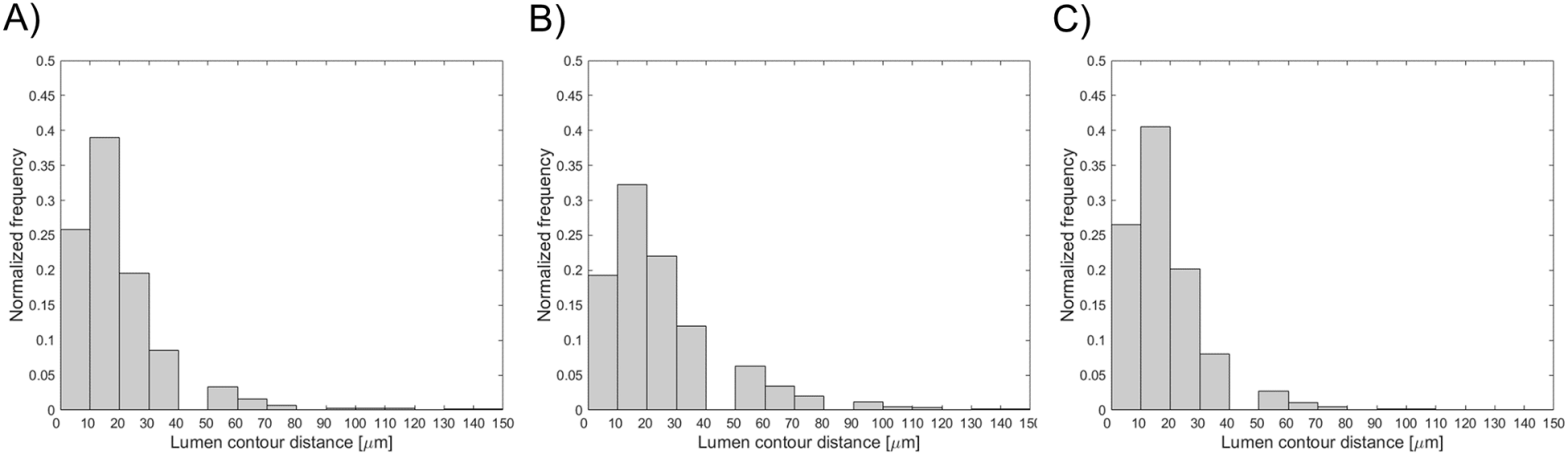
Distribution of the distance between the lumen contours obtained on 160 randomly selected OCT images with (A) the automatic algorithm and manual segmentation by image reader 1, (B) the automatic algorithm and manual segmentation by image reader 2, and (C) the two manual segmentations.

The distribution of the distance between the lumen contours obtained with the automatic and manual segmentation methods is displayed in Fig 8. The 25^th^, 50^th^, and 75^th^ percentiles are listed in Table 2.

### 3.2 Stent struts detection

Table 4 reports the 25^th^, 50^th^, and 75^th^ percentiles of the distributions of number of struts per OCT frame, identified with the automatic and manual segmentations. No significant differences between the number of struts detected with the different methods were found (chi-square = 5.9, p = 0.0523).

**Table 4:** Percentiles of the distributions of number of struts obtained with the automatic and manual segmentation methods, and total and radial distances between the centroid of each automatically segmented strut and the nearest manually identified strut.

	25^th^ percentile	50^th^ percentile	75^th^ percentile
**Number of struts**			
**Auto**	11	13	15
**R1**	11	14	15
**R2**	10	12	15
**Total distance between struts [μm]**			
**Auto vs. R1**	18.38	29.07	41.11
**Auto vs. R2**	18.38	29.07	46.87
**R1 vs. R2**	13.00	13.00	26.00
**Radial distance between struts [μm]**			
**Auto vs. R1**	4.58	9.64	15.73
**Auto vs. R2**	5.31	11.30	18.37
**R1 vs. R2**	2.61	9.58	14.37

Auto—automatic detection algorithm; R1 –image reader 1; R2 –image reader 2

**Fig 9:**
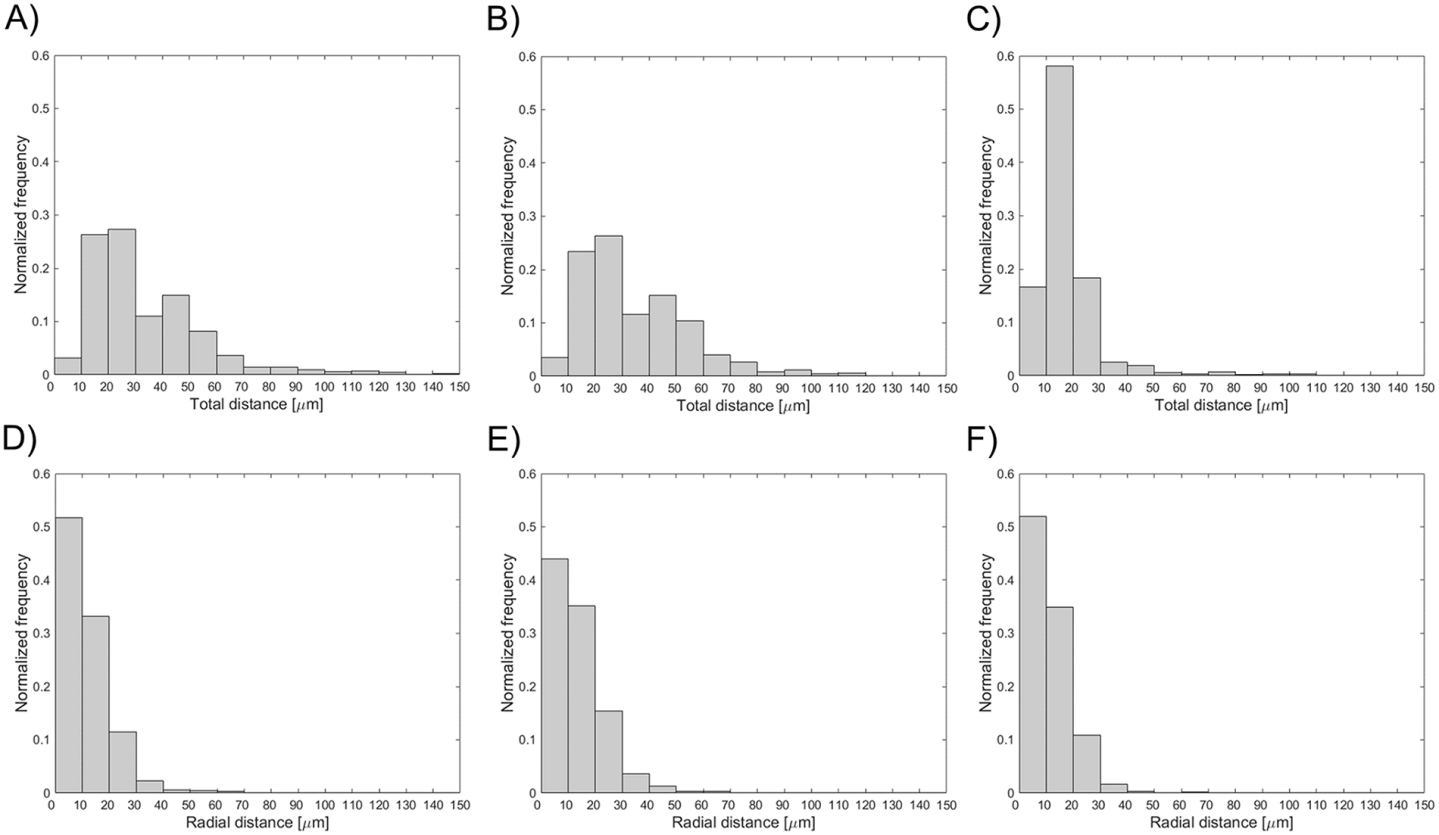
Distributions of the total (top) and radial (bottom) distances between the centroid of each segmented strut (A, D) by the automatic algorithm and the nearest manually identified strut by image reader 1, (B, E) by the automatic algorithm and the nearest manually identified strut by image reader 2, and (C, F) by the two manual segmentations.

**Fig 10:**
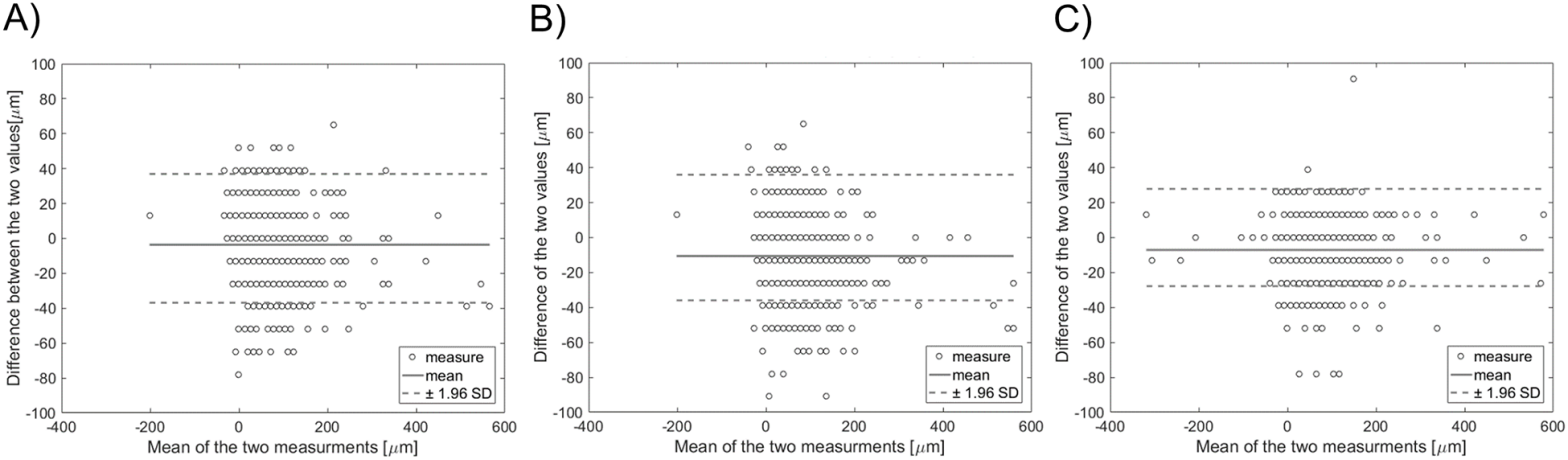
Bland-Altman diagrams of length of apposition (LOA): A) automatic segmentation against R1; B) automatic segmentation against R2; C) manual segmentation by R1 against R2.

The similarity indexes are reported in Table 3. The distributions of the total and radial distances between the centroid of each automatically segmented strut and the nearest manually identified strut are shown in Fig 9. The 25^th^, 50^th^, and 75^th^ percentiles of these distributions are reported in Table 4. The Bland-Altman diagrams of LOA are displayed in Fig 10. The 95% confidence range in the LOA differences was between -41.30 μm and 34.00 μm, -47.27 μm and 26.13 μm, and -35.12 μm and 20.96 μm for the automatic algorithm versus R1, the automatic algorithm versus R2, and R1 versus R2, respectively.

**Fig 11:**
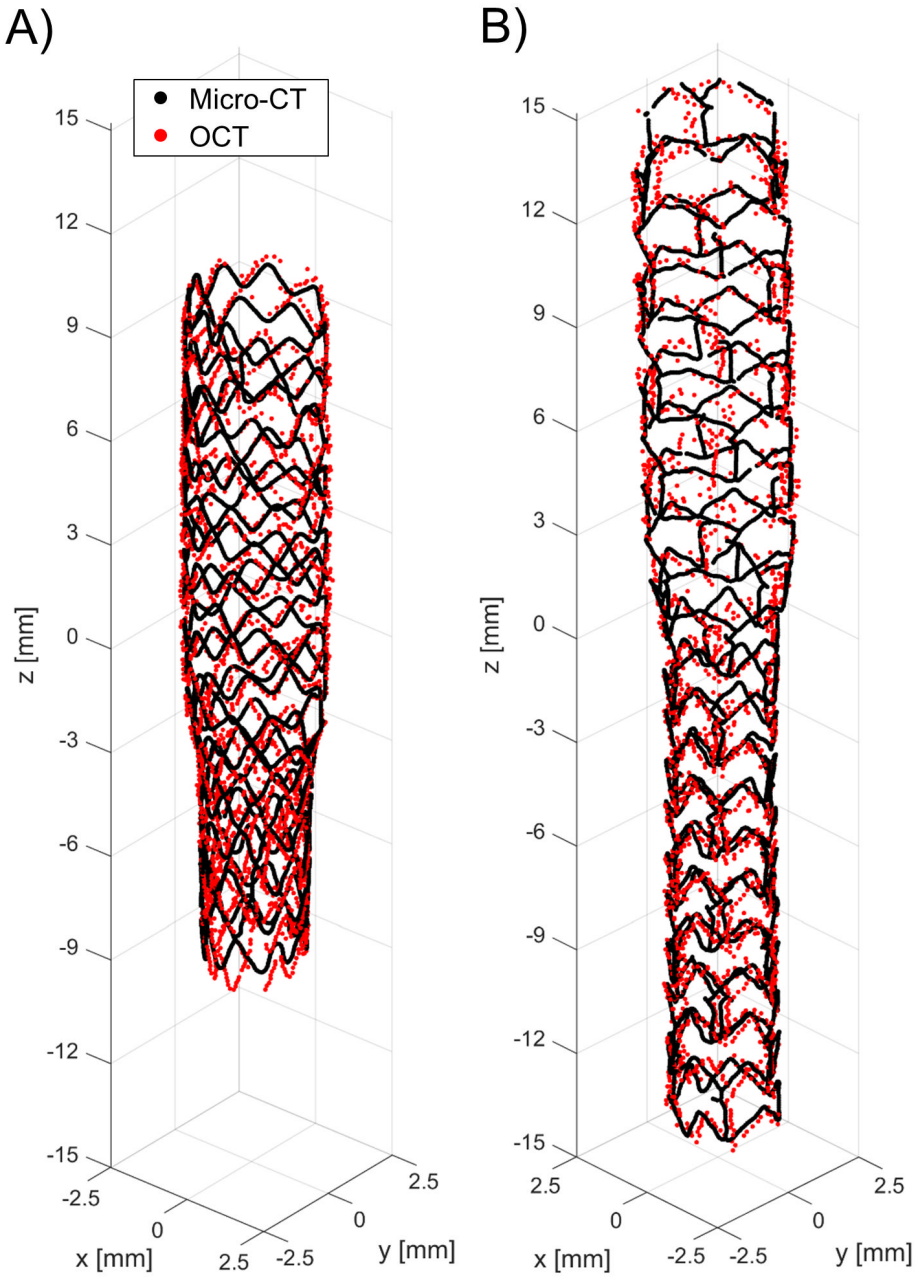
Superimposition of the stent point clouds obtained through the automatic detection algorithm (red) and micro-CT (black): A) Case 1 (Resolute Integrity 3x18 mm). B) Case 2 (Xience Prime 3x28 mm).

**Fig 12:**
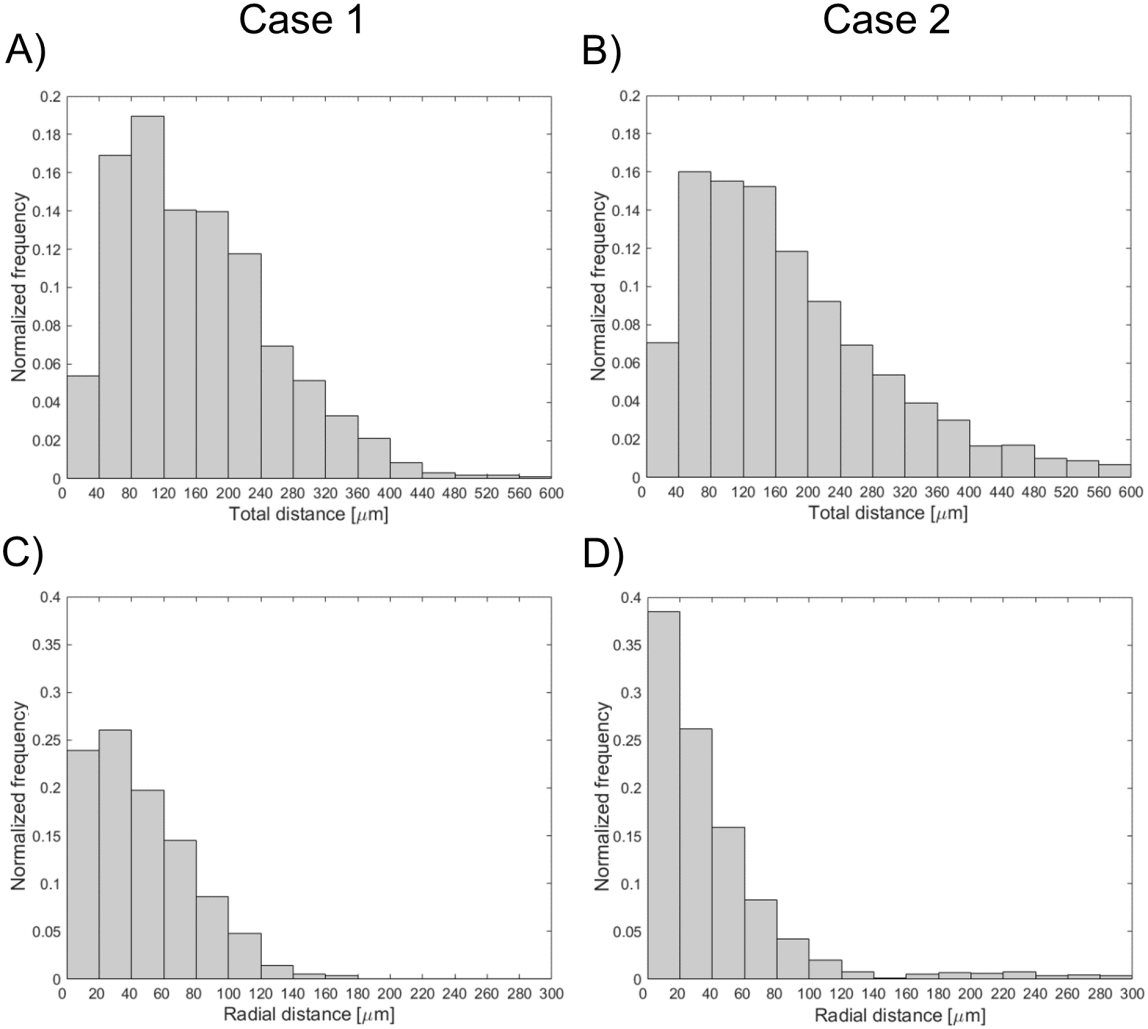
Distributions of the total (top) and radial (bottom) distances between corresponding points of the stents: A, C) Case 1 (Resolute Integrity 3x18 mm). B, D) Case 2 (Xience Prime 3x28 mm).

Fig 11 shows the superimposition of the stent point clouds obtained for cases 1 and 2 by applying the stent struts detection algorithm with the centerline points of the corresponding stents reconstructed from micro-CT after the registration process. Qualitatively, a good agreement between the point clouds of the two investigated stents was found. The distribution of the total and radial distances between corresponding points of the stents are presented in Fig 12. The 25^th^, 50^th^, and 75^th^ percentiles of the total distances were 85.43 μm, 149.22 μm, and 244.59 μm, respectively, for case 1, and 87.07 μm, 148.64 μm, and 244.22 μm, for case 2. The 25^th^, 50^th^, and 75^th^ percentiles of radial distances were 18.45 μm, 39.56 μm, and 68.07 μm, respectively, for case 1, and 18.89 μm, 40.20 μm, and 69.18 μm, for case 2.

### 3.3 Repeatability of the OCT pullback

Table 5 reports the lumen volume and the mean number of struts per frame obtained for the seven repetitions of the OCT scan of Case 3. The 25^th^, 50^th^, and 75^th^ percentiles of the distributions of the lumen volume were 358.51 mm^3^, 360.41 mm^3^, and 365.56 mm^3^ while those of the distributions of mean number of struts per frame were 11.95, 12.56, and 13.29.

**Table 5:** Lumen volume and the mean number of struts per frame obtained for the seven repetitions of the OCT scan of Case 3.

OCT scan repetition	1	2	3	4	5	6	7
**Lumen volume [mm**^**3**^**]**	366.56	358.29	360.41	359.18	377.77	362.55	356.16
**Mean number of struts per frame**	12.56	10.96	11.83	13.47	14.74	12.39	12.72

### 3.4 Applicability to patient-specific cases

**Fig 13:**
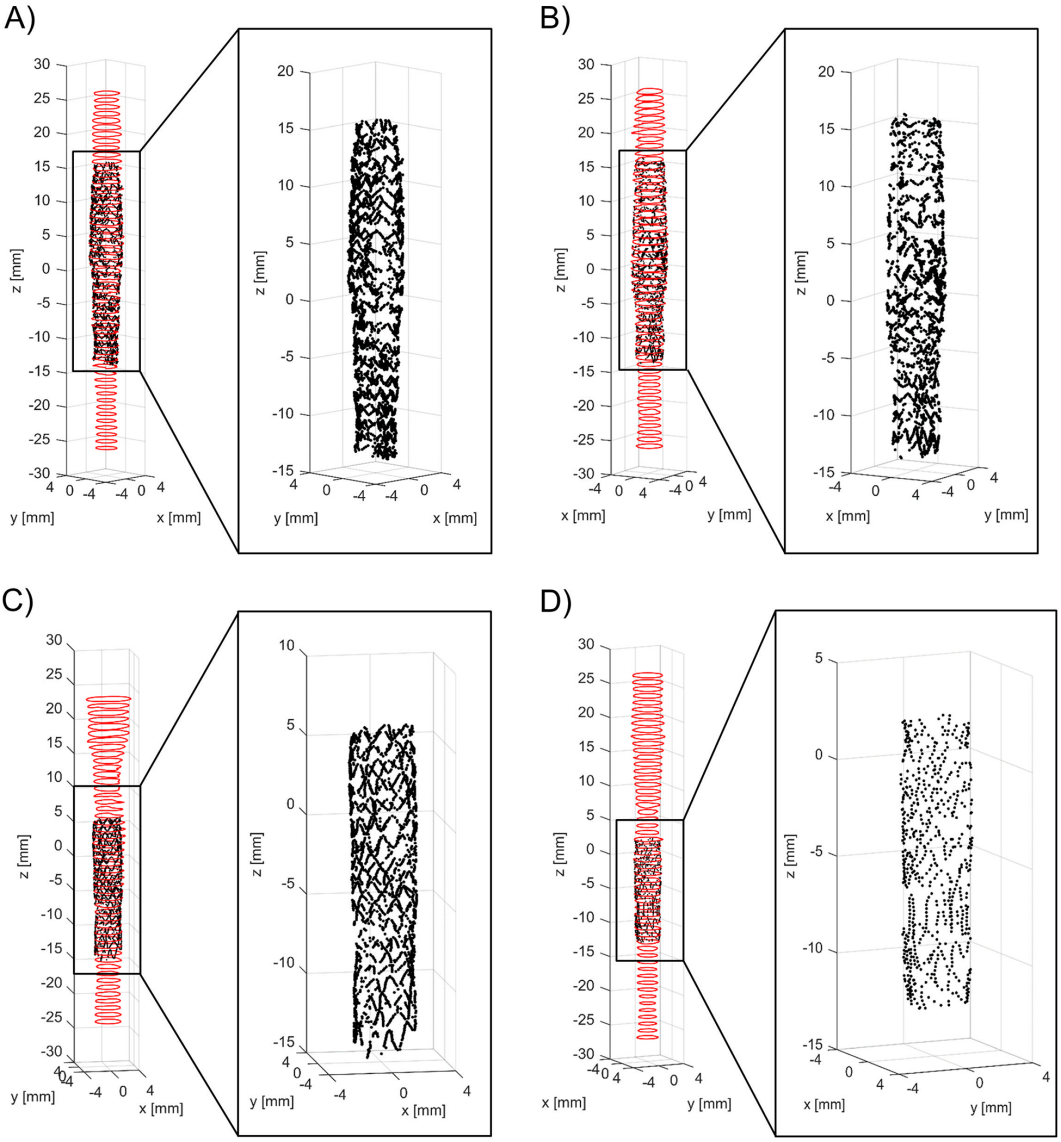
Three-dimensional lumen and stent point clouds of the four patient-specific stented coronary segments under investigation, which were obtained by applying the developed lumen border and stent struts detection algorithms: A) distal right coronary artery segment treated with Xience Prime 3.5x28 mm; B) mid right coronary artery segment treated with Xience Prime 3.5x28 mm; C) left anterior descending coronary artery segment treated with Resolute Integrity 3.5x18 mm; D) left anterior descending coronary artery segment treated with Resolute Integrity 2.75x14 mm. For each case, details of the stent point cloud are provided.

In Fig 13 the lumen contour and stent struts point clouds of the four investigated *in vivo* cases are depicted. As shown by the figure, both lumen contours and stent struts were successfully identified by the segmentation method. Furthermore, in all cases the stent design is clearly recognizable.

## 4. Discussion

Nowadays, OCT is successfully used for the assessment of atherosclerosis in coronary arteries and the evaluation of stenting procedures during intervention and at follow-up [1–3]. Commercially available OCT systems allow one to acquire images at high frame rate (up to 200 frames per second), resulting in a large number of cross-sectional images per pullback (e.g. 500 or more). Manual analysis of these large OCT datasets for the detection of lumen contours and stent struts is very time-consuming and unsuitable for real-time applications [3]. In this study, an automatic method was developed for segmentation and 3D visualization of both vessel lumen contours and stent struts. The method was applied to coronary bifurcation phantoms for validation purposes.

Validation of the lumen contour detection algorithm against manual segmentation gave good results. The lumen areas that were calculated through the automatic method and manually by the image readers were not statistically different. The 95% confidence range in the lumen area percentage differences was comparable with that obtained in other studies for *in vivo* OCT datasets. In particular, the limits of agreement were lower than those of Celi and Berti (differences between -1.2 mm^2^ and 1.2 mm^2^) [12] and Chatzizisis and colleagues (differences between -1.60 mm^2^ and 1.30 mm^2^) [11], but slightly higher than those of Sihan and colleagues (percentage differences between -3.20 and 4.00%) [21]. The good efficacy of the lumen detection algorithm was also demonstrated by the similarity indexes, with mean value higher than 95% and low standard deviation. Additionally, the distributions of the distances between the lumen contours obtained with the automatic and manual segmentation methods (Fig 8A and 8B) were skewed to the left with the 75^th^ percentile equal to 26 and 39 μm, i.e. only twice and three times the pixel size, respectively.

Good results were obtained from the validation of the stent struts detection algorithm against manual segmentation. No statistically significant differences in the number of detected struts were found between the automatic detection algorithm and the manual segmentations. Similarity indexes were good, with mean values higher than 85%. Their standard deviation was higher than that obtained for the lumen detection, suggesting a higher variability of the segmentation quality between the different images. The sensitivity of the struts detection algorithm was similar to that found by Wang and colleagues (mean value of 94%) by analyzing *in vivo* OCT datasets [24]. The distribution of the distance between the centroid of each automatically segmented strut and the nearest manually identified strut (Fig 9) showed that ~90% of the automatically detected struts had a radial distance lower than 50 μm, which approximately corresponds to half thickness of the strut of both Resolute Integrity and Xience Prime stents. Furthermore, the limits of agreement for the LOA differences were similar to those obtained by Ughi and collaborators for *in vivo* OCT images (LOA differences approximately between -40 μm and 40 μm) [17].

The application of the proposed detection method to *in vitro* OCT images enabled us to compare the 3D stent point clouds of two selected cases with the corresponding micro-CT scans, which were considered as reference. The two cases were characterized by different stents, i.e. Resolute Integrity and Xience Prime. In particular, the Resolute Integrity stent is formed from a single Cobalt-Chromium wire bent into a continuous sinusoid pattern and has struts with circular cross-section. The Xience Prime is laser cut from Cobalt-Chromium tubes and has struts with rectangular cross-section. The comparison showed a good qualitative agreement between the point clouds of both investigated stents (Fig 11), thus demonstrating that the detection method can be successfully applied to different stent designs. Quantitative comparison between the corresponding stent points resulted in promising results, with median values of ~150 μm and ~40 μm for the total and radial distances of both cases, respectively. This comparison is affected by different factors, which may result in over-estimated distance values. In particular, in this study the ICP optimization algorithm was used as registration method. However, other registration algorithms, such as those based on genetic algorithms optimization [38], may result in better results. Additionally, the uncertainties related to the 3D stent reconstruction and centerline extraction from micro-CT have to be taken into account.

In the current study, the repeatability of the detection algorithms was also investigated by calculating the lumen volume and the mean number of detected struts per frame for seven repeated OCT scans of one case. Results showed limited statistical dispersion of values for the analyzed quantities around the median. Such dispersion might be partially addressed to the impossibility of imposing a unique starting point for the OCT pullback among all performed acquisitions.

To demonstrate the applicability of the developed method to *in vivo* OCT pullbacks, the detection algorithms were applied to four patient-specific cases of diseased coronary artery segments treated with two different stents (i.e. Xience Prime and Resolute Integrity). Results showed that the algorithms were able to detect both lumen and stent struts in all cases (Fig 13). Indeed, both *in vitro* and *in vivo* OCT images share the same main features (e.g. stent struts appearing as high intensity region followed by a trail shadow) that provide information to correctly identify lumen contours and stent struts.

The proposed detection algorithms can be used in different applications, including quantification of lesion severity and the evaluation of stent strut malapposition [3]. The algorithms require ~5 minutes for the detection of lumen contours and stent struts of one OCT dataset on a desktop computer equipped with CPU i7-950 @3.07 GHz and 16 GB of RAM. The processing time can be dramatically reduced by converting the Matlab code to a lower level language (e.g. C++) [12] and by using graphics processing units (GPU) for the calculations [9], thus allowing for on-line applications. The output of the proposed algorithms can be also used as a starting point for the reconstruction of 3D models of (stented) coronary arteries and subsequent computational fluid dynamics simulations, which allow investigation of the local hemodynamics. Indeed, the implantation of a stent in a coronary artery alters the physiological blood flow and induces recirculation and low, oscillatory endothelial shear stresses that may lead to in-stent restenosis [39,40]. In several recent studies, hemodynamic simulations were performed in 3D geometries of coronary arteries, which were reconstructed by coupling the lumen contours, automatically detected from OCT images, with the vessel centerline, extracted from angiography or computed tomography [41–46]. However, both vessel lumen and stent geometries were reconstructed starting from OCT images for subsequent hemodynamic analyses only in few studies [47,48]. In these works, different strategies for volumetric reconstruction of the stent from the stent points cloud identified from OCT were proposed with limited success. Future applications of our detection algorithm will be applied in this context in order to automatically reconstruct the 3D geometry of stented coronary bifurcations for subsequent fluid dynamics analyses. Fusion of OCT with angiography or computed tomography will be necessary to capture the correct orientation in the 3D space of the stented vessel [45,49].

Although the present study showed promising results, limitations are present. Our stent strut detection algorithm does not work in case of polymeric bioresorbable stents—such as the Absorb BVS (Abbott Vascular)—because the trail shadows, caused by metallic struts and allowing stent detection, are absent in the OCT images. The creation of the lumen and stent point clouds might be affected by the relative axial twist between OCT frames. This error was not reduced because of the lack of landmarks in the acquisitions and negatively influenced the comparison between the 3D stent point clouds from OCT with the corresponding micro-CT scans.

## 5. Conclusions

This study presents a robust automatic method for detection and 3D visualization of both vessel lumen contours and stent struts of stented coronary arteries. The method was initially applied to *in vitro* OCT images, which were acquired in stented coronary bifurcation phantoms. Validation against manual segmentation gave good results with high values for similarity indexes for both lumen contours and stent struts algorithms. The comparison between the 3D stent point clouds obtained from OCT of two selected cases with the corresponding micro-CT scans showed a good qualitative agreement. The quantitative comparison between the corresponding OCT and micro-CT reconstructions resulted in acceptable differences in terms of distances. Furthermore, the repeatability of the detection algorithms was analyzed, resulting in repeatable values of lumen volume and mean number of struts per frame. Finally, the applicability of the detection method to *in vivo* OCT images was successfully demonstrated by identifying lumen contours and stent struts in four patient-specific cases of stented coronary arteries.
